# Supplementary material for: Revealing semantic and emotional structure of suicide notes with cognitive network science
Source: Sci Rep. 2021 Sep 30;11:19423. doi: 10.1038/s41598-021-98147-w (PMC8484592; doi:10.1038/s41598-021-98147-w)
Supplement: Supplementary file 1 — Supplementary Information. [file 41598_2021_98147_MOESM1_ESM.pdf]

Revealing semantic and emotional structure of suicide notes with cognitive network science - Supporting Information

Andreia Sofia Teixeira[1,2,3,+], **Szymon Talaga**[4,+], **Trevor James Swanson**[5], **and Massimo Stella**[6,7,*]

[1]INESC-ID, R. Alves Redol 9, 1000-029 Lisboa, Portugal
[2]Indiana Network Science Institute, Indiana University, 1001 IN-45 Bloomington IN, USA
[3]Hospital da Luz Learning Health, Luz Saúde, Avenida Lusíada, 100, Edifício C, 1500-650 Lisboa, Portugal
[4]University of Warsaw, The Robert Zajonc Institute for Social Studies, Stawki 5/7, Warszawa 00-183, Poland
[5]University of Kansas, Department of Psychology, 1415 Jayhawk Blvd, Lawrence KS, 66045, USA
[6]CogNosco Lab, Department of Computer Science, University of Exeter, Exeter EX4 4PY, UK
[7]Complex Science Consulting, Via Amilcare Foscarini 2, 73100 Lecce, Italy
[+]Andreia Sofia Teixeira contributed equally to this work with Szymon Talaga.
[*]Corresponding author: massimo.stella@inbox.com

Network Construction

Two types of networks were adopted for the current analysis:

- Co-occurrence (CO) network in which nodes represent concepts and links indicate succession relationships. Thus, relationships were captured through a sequential chain, establishing links between preceding and subsequent words. For instance, the sentence "The pen is red" would feature links "the–pen", "pen–is" and "is–red". This straightforward approach is reminiscent of word co-occurrence and can capture succession relationships in language, e.g. a word specifying semantic features of another one.

- Undirected, weighted network of relationships induced by SVO triplets extracted from the notes. Each SVO triplet was decomposed into three possible links included in a triple:

  1. subject–verb
  2. verb–object
  3. subject–object

  For instance, the triple "he–look–at" consists of the following three pairs: (1) "he–look", (2) "look–at", and (3) "he–at". Edge weights are equal to the number of co-occurrences of two words in the same SVO triplets. Note that this way of decomposing SVO triplets into node pairs does not introduce any structural bias, as each component (subject, verb and object) appears exactly two times over three pairs generated from a single triplet. On the other hand, tokens playing central syntactic roles (i.e. subject and verbs) will appear in more SVO triplets than less central ones. Therefore, these tokens will have higher degrees and strengths (sums of edge weights) in an SVO network. This is an important property of our method which encodes some of the crucial semantic features of text corpora directly into degree/node strength distributions.

  In order to filter out errors and relationships between words that were accidental and/or idiosyncratic for individual suicide notes, the SVO network was limited only to relations (edges) with weights equal to or greater than 2. In other words, only relationships that occurred at least twice over the entire corpus were considered. Then, all positive weights were decreased by 1 to remove the truncation of the lower tail at 2 in order to make sampling from the appropriate canonical ensemble (undirected weighted configuration model[1]) more feasible.

The above linguistic networks were also enriched with:

- sentiment labels, i.e. positive/negative/neutral, indicating the overall pleasantness of a concept as expressed by large audiences in psycholinguistic studies about common language. These labels identified words in the lower quartile (negative), middle quartiles (neutral) and upper quartile (positive) of valence scores as obtained from[2];
- emotional labels, expressing the emotions elicited by a given word as indicated by large audiences in psycholinguistic studies about common language. The considered emotions were those from the NRC Emotion Lexicon[3], namely anger, anticipation, fear, disgust, joy, sadness, surprise and trust.

Both the above sentiment and emotional datasets are relative to overall, global perceptions of concepts as represented in mainstream populations. Hence, these datasets are not directly informative about the subjective emotional perceptions portrayed by people who committed suicide. In order to reconstruct these subjective perceptions, network measures are required. Moreover, we performed a sensitivity analysis on the valence scores, using tertiles instead of quartiles, since this different distribution could potentially impact the results of Study 1. That was note the case. This new division of negative, neutral and positive words, revealed no significant change in the emotional balance analysis, resulting in similar values for degree of balance (98%) and for triads distribution (0.02%, 0.14%, 0%, 0.84%).

The CO and SVO networks enable the reconstruction of a semantic frame in terms of a network neighborhood of a given concept. As an example, the semantic frame of "love" is represented by the first neighbors of concepts syntactically related to "love" by people who committed suicide in their suicide notes. Checking this semantic frame/network neighborhood provides crucial information about the contexts and perspectives that featured "love" in the suicide notes. On the emotional level, sentiment and emotional attributes are not universal, as they could change according to the subjective perceptions of the authors or the context in which concepts appear. For instance, "love" is usually indicated as a positive word bringing emotions of trust and joy. But what about associations like "betraying love" or "missing love"? Placing "love" in different contexts can alter its subjective perception. This is the main reason why language cannot simply be considered as a bag of words, i.e. a collection of isolated concepts, but rather as a network of interconnected linguistic units whose meaning and emotions can change according to the way they are networked together. Therefore the above networks provide access to the associative and emotional perceptions of conceptual entities in the minds of people committing suicide.

Additional natural language processing

Words in all notes were lemmatized and annotated with part-of-speech and dependency tags as classified with a state-of-the-art NLP library *Spacy*[4] based on OntoNotes (v5) annotated corpus and Penn Treebank[5]. The tags specify roles played by particular words in a sentence as well as syntactic dependency relationships between them. This additional information was used to derive network representations of the notes capturing more fundamental syntactic links instead of simpler, sequential relationships between preceding and subsequent words. Specifically, it allowed a decomposition of all sentences into a kind of generalized subject-verb-object (SVO) triplets. Here, an SVO triplet consists of a subject, which is seen as an active agent, a verb seen as an action performed by the subject, and an object standing for anything that the action (verb) performed by the subject relates to. This is why the decomposition used here is somewhat more general, as any token other than nominal subject and subordinate to a verb in a syntactic tree of a sentence is considered an object.

For instance, in our approach the following sentence is decomposed into four different SVO triplets:

- He was looking at a tree, which was very tall.
    1. he–look–at
    2. he–look–tree
    3. tree–be–very
    4. tree–be–tall

As the example shows, the method disaggregates relative clauses such as "tree, which is tall" into separate SVO triplets. This way it is more capable of capturing the semantics of compound and complex sentences. Moreover, more important meaning making tokens (such as "he", "look" and "tree" in the example above) appear in multiple triplets by design. Because of this, even simple summaries such as frequencies of words over all SVO triplets can capture important semantic features of a text corpora.

The second important processing step was a custom lemmatization which accounted for the specific way in which the suicide notes were anonymized. Most words were lemmatized according to the standard rules implemented for the English language in *Spacy*[4]. However, all names of persons in the notes were substituted with several generic placeholder names, such as Jane or William, and so were reduced to a special "s/he" lemma. Moreover, all occurrences of "he" and "she" were also lemmatized this way.

Extracting SVO triplets

The general procedure used for extracting SVO triplets is relatively straightforward. First, documents are tokenized into sentences and sentences are tokenized into words. A word is considered **semantic** if it is a (proper) noun, pronoun, verb, adverb, adjective, adposition or a negation modifier (not). Then, all semantic words are assigned with one of the following classes:

- `SUBJECT`. Words which are either active or passive nominal or clausal subjects (according to syntactic dependency tags).
- `VERB`. Words which are either verbs (according to part-of-speech tags) or relative clauses (according to syntactic dependency tags).
- `OBJECT`. Any semantic word which is neither a verb nor a subject.

Additionally, we define two procedures.

- `GET_VERB(token)`.
  Return nearest `VERB` word which is above `token` in the syntactic dependency tree.
- `GET_SUBJECT(token)`.
  Define `verb = GET_VERB(token)`.
  Define `subj` to be the nearest `SUBJECT` word below `verb` in the syntactic dependency tree.
  If `subj` is a (proper) noun according to its POS tag, return `subj`.
  Otherwise, return the nearest `NOUN` word above `subj` in the syntactic dependency tree.

Finally, SVO triplets are extracted from a sentence according to the following procedure:

- For each `word` in the sentence:
  - If `word` is `OBJECT`:
    - Return a triplet: `GET_SUBJECT(word)`, `GET_VERB(word)`, `word`.

For instance, in the example sentence "He was looking at a tree, which was very tall" there are four `OBJECT` words which are mapped to four SVO triplets:

1. `at` ↦ `GET_SUBJECT(at), GET_VERB(at), at` ↦ `he, look, at`
2. `tree` ↦ `GET_SUBJECT(tree), GET_VERB(tree), tree` ↦ `he, look, tree`
3. `very` ↦ `GET_SUBJECT(very), GET_VERB(very), very` ↦ `tree, be, very`
4. `tall` ↦ `GET_SUBJECT(tall), GET_VERB(tall), tall` ↦ `tree, be, tall`

Structural balance theory

Structural balance theory, first explored by Heider[6], states that for a signed triad to be balanced the product of its signs must be positive. Thus, from the four possible triads – $\{+,+,+\}$, $\{-,+,+\}$, $\{-,-,+\}$, $\{-,-,-\}$ – only the first and third are considered balanced. As an example, if we think about the following statements "a friend of my friend is my friend", "an enemy of my enemy is my friend", along with similar others, we are able to verify that they follow the concept of balance as defined by Heider.

Some years later, Cartwright and Harary generalized the concept of structural balance to social networks, introducing signed graphs in which edges had positive and negative signs corresponding to positive or negative ties between individuals[7,8]. They extended the concept of balanced triads to balanced networks by allowing cycles with more than three edges. A cycle is considered balanced if the product of the signs of its edges is positive, i.e., if there are no odd number of negative edges in a cycle. To measure structural balance, Harary introduced the concept of *Degree of Balance* (DoB) of a signed network as the ratio of the number of positive cycles to the total number of cycles [9]. Let G be a signed graph, $c(G)$ be the number of cycles of G, $c_+(G)$ be the number of positive cycles of G, and $DoB(G)$ be the degree of emotional balance of G. Then:

$$DoB(G) = \frac{c_+(G)}{c(G)}. \tag{1}$$

In this work we use cycles of size three – triads.

We checked that the deletion of stopwords did not impact either emotional balance nor the fractions of triadic closure in any significant way, i.e. it produced changes in the considered fractions below 2%.

Semantic network distance and word prominence

The identification of semantically related concepts is traditionally performed by semantic latent analysis, which maps the problem of measuring conceptual distance into selecting appropriate metrics in a vectorial space of words. However, in predicting semantic relatedness, semantic latent analysis was recently outperformed by network distance in cognitive networks, i.e. counting the smallest number of conceptual links connecting any two concepts in the same connected component of a given network[10]. We build upon this evidence and define conceptual relatedness as concepts being at a shorter network distance. A concept which is related to, i.e. at shorter network distance from, almost all other concepts must be prominent. This intuitive definition of conceptual prominence is methodologically implemented by closeness centrality[11], which identifies concepts that are at shorter network distance from all other connected concepts.

Closeness centrality has been successfully used in previous investigations as a proxy of conceptual prominence predicting word acquisition (cf.[12]). Also in the current analysis, we use closeness centrality as a quantitative way for identifying prominent concepts in the reconstructed mindset around suicidal ideation.

As a statistical baseline, the closeness centrality of concepts in the empirical networks was matched against closeness centrality in configuration models[11], i.e. random networks fixing the empirical degrees of words but otherwise randomizing conceptual links. We checked that the deletion of stopwords did not alter the ranking based on closeness centrality in the co-occurrence network.

Rank	Co-occ. Netw.	Free Asso.	Co-occ. Restr.
1	be	time	be
2	have	money	have
3	to	love	to
4	and	work	and
5	of	good	of
6	love	sad	love
7	for	food	do
8	do	home	for
9	in	day	in
10	take	life	take
11	go	happy	go
12	get	out	get
13	way	death	make
14	one	place	live
15	make	water	way
16	live	child	one
17	help	help	help
18	want	person	want
19	time	fun	time
20	day	bad	work
21	call	letter	day
22	end	me	end
23	life	car	all
24	thing	man	life
25	work	blue	call
26	all	house	not
27	not	red	feel
28	feel	now	thing
29	family	book	this
30	give	save	give

Table 1. Top 30 concepts ranked in descending order based on their closeness centrality in the original co-occurrence network (Co-occ. Netw.), the baseline network of free associations (Free Asso.) and the original co-occurrence network restricted to the same concepts featured in the free association network (Co-occ. Restr.).

SI Table 1 reports the most prominent concepts, based on closeness centrality, in the original network of co-occurrences (with 2075 words) and the baseline free association network (with 1577 words, a subset of words in the co-occurrence network). As an additional check, words are ranked also in the subgraph of the co-occurrence network featuring only those words present

in the free association network. Co-occurrences in suicide notes reflect a structural organisation of knowledge where love is more central than other topics, a pattern that is not observed in the baseline free association dataset, which features ”time” and ”money” as concepts with a higher closeness to all other connected words. Even by performing node alignment between the co-occurrence and the free associations networks, i.e. considering a subgraph of co-occurrences only between words present in the free association network, ”love” remains more central than other concepts. These results indicate that ”love” in the organisation of knowledge as assembled by authors of suicide notes was more central than expected in the knowledge of mindwandering as represented by free associations.

Network degeneracy

Degeneracy of a network[13] measures the tendency that a random walker starting from a random node after one step ends up in a limited set of central nodes. Let $\mathbf{W}$ be a normalized weighted and undirected adjacency matrix such that weights in each row $\mathbf{W}_{i\bullet}$ sum up to 1 so they can be interpreted as probability distributions over the next position of a random walker starting at node i. Then, degeneracy of the graph represented by $\mathbf{W}$ is defined as a normalized difference between maximal and observed entropy of the probability distribution $\mathbf{W}_{i\bullet}$ averaged over all nodes $i = 1, \ldots, N$ assuming they are equally likely:

$$degeneracy = \frac{\log_2 N - H(\langle \mathbf{W}_{i\bullet} \rangle)}{\log_2 N - S} \in [0, 1] \tag{2}$$

where $\langle \mathbf{W}_{i\bullet} \rangle$ is the vector of column means of $\mathbf{W}$, $\langle \mathbf{W}_{i\bullet} \rangle = 1/N \sum_{i=1}^{N} \mathbf{W}_{i\bullet}$, and $H(\cdot)$ is Shannon entropy. The S term is $H(\langle \mathbf{W}_{i\bullet} \rangle)$ of an undirected star graph with N nodes, that is:

$$S = -\frac{N-1}{N} \log_2 \left(\frac{N-1}{N} \right) - \frac{1}{N} \log_2 \left(\frac{1}{N} \right) \tag{3}$$

Emotional Profiling

Emotional profiling was performed by labeling words according to the emotion they elicit, as indicated in the NRC emotion lexicon[3]. The dataset included 8 basic emotional states, whose combination can give rise to a wide variety of nuanced emotion. Emotional profiling was performed as in previous studies[14], considering the number of words eliciting a given emotion in a certain network region, e.g. in the network neighborhood of a certain concept. The emotional profile of a word p was considered by counting the fraction $f_i(p)$ of words syntactically linked to w eliciting emotion i. By definition $f_i(p)$ ranges between 0 (no conceptual associates of p elicit emotion i) to 1 (all concepts linked to p elicit emotion i). As a reference model, we used a random sampling of words fixing the empirical sample size, e.g. the number of words syntactically linked to p, but neglecting empirical syntactic associations. Counting the fraction of randomly sampled words eliciting emotion i provided direct-sampling distributions of random emotional profiles. We used these random distributions in order to attribute a z-score to the observed emotional profile $f_i(p)$. This statistical procedure, with a significance level fixed at $\alpha = 0.05$, enabled a comparison of the strength of emotions elicited by individual concepts in our considered networks.

On the one hand, the visualization of z-scores facilitates the immediate understanding of which were the stronger emotional intensities elicited by a given concept. On the other hand, the comparison provides additional information about how rich a concept can be in associations eliciting a given emotion.

Notice that stopwords did not possess any emotional data and thus did not influence the emotional profiles reported in the main text.

Sentence examples

Table 3 presents a set of examples of sentences consisting of (undirected) relations between several selected concepts.

Rank	Word	z-score	Significant?	Rank drop	Rank	Word	z-score	Significant?	Rank drop
1	love	2.065	Yes	6	21	feel	0.389		6
2	do	1.134		2	22	family	1.220		116
3	take	0.884		6	23	give	0.113		2
4	go	1.027		6	24	tell	0.222		-3
5	get	0.780		4	25	try	0.733		17
6	way	1.328		20	26	know	0.318		-6
7	one	1.424		33	27	start	1.408		55
8	make	0.691		8	28	good	0.988		33
9	live	1.197		30	29	as	0.193		-3
10	help	1.261		20	30	friend	1.174		82
11	want	0.782		9	31	year	0.865		43
12	time	1.339		33	32	money	0.997		45
13	day	1.217		54	33	leave	0.530		13
14	call	1.016		16	34	hope	0.797		30
15	end	1.278		53	35	child	1.281		121
16	life	1.259		61	36	think	0.648		34
17	thing	1.224		28	37	use	1.259		102
18	work	1.274		38	38	other	1.077		146
19	all	0.306		4	39	mother	1.205		129
20	not	3.102	Yes	-13	40	come	0.244		-5

Table 2. Top 40 concepts based on closeness centrality in the CO network. To detect how empirical syntactic relationships contributed to closeness centrality, 1000 configuration models with random relationships were built. Empirical concept centrality was compared against mean random expectation, enabling a measurement of the mean rank drop due to randomizing conceptual links. "Love" was found to be 6 positions higher in the empirical ranking than in random configuration models and was the only one among the top words for which rank drop was statistically significant.

References

1. Squartini, T., Mastrandrea, R. & Garlaschelli, D. Unbiased sampling of network ensembles. *New J. Phys.* **17**, 023052, DOI: 10.1088/1367-2630/17/2/023052 (2015).
2. Warriner, A. B., Kuperman, V. & Brysbaert, M. Norms of valence, arousal, and dominance for 13,915 english lemmas. *Behav. research methods* **45**, 1191–1207 (2013).
3. Mohammad, S. M. & Turney, P. D. Crowdsourcing a word–emotion association lexicon. *Comput. Intell.* **29**, 436–465 (2013).
4. Honnibal, M., Montani, I., Van Landeghem, S. & Boyd, A. spaCy: Industrial-strength Natural Language Processing in Python, DOI: 10.5281/zenodo.1212303 (2020).
5. Weischedel, R. *et al.* Ontonotes release 5.0 ldc2013t19. *Linguist. Data Consortium, Philadelphia, PA* **23** (2013).
6. Heider, F. Attitudes and cognitive organization. *J. Psychol.* **21**, 107–112 (1946Journal of Psychology).
7. Harary, F. On the notion of balance of a signed graph. *Mich. Math. J.* **2**, 143–146 (1953).
8. Cartwright, D. & Harary, F. Structural balance: a generalization of heider's theory. *Psychol. review* **63**, 277 (1956).
9. Harary, F. On the measurement of structural balance. *Behav. Sci.* **4**, 316–323 (1959).
10. Kenett, Y. N., Levi, E., Anaki, D. & Faust, M. The semantic distance task: Quantifying semantic distance with semantic network path length. *J. Exp. Psychol. Learn. Mem. Cogn.* **43**, 1470 (2017).
11. Newman, M. *Networks* (Oxford university press, 2018).
12. Stella, M., Beckage, N. M. & Brede, M. Multiplex lexical networks reveal patterns in early word acquisition in children. *Sci. reports* **7**, 46730 (2017).
13. Klein, B. & Hoel, E. The Emergence of Informative Higher Scales in Complex Networks. *Complexity* **2020**, 1–12, DOI: 10.1155/2020/8932526 (2020).
14. Stella, M. Text-mining forma mentis networks reconstruct public perception of the stem gender gap in social media. *arXiv preprint arXiv:2003.08835* (2020).

Note id	Concepts	Sentences
37SA	love-you	(...) last night I know **you do love** me. (...) I **love you you** stupid head (...) I **love you** mush pleace rember all the **love I have shown you**. (...)
99SA	go-i	(...) **I could go** into detail explaining why I feel that way (...) **Im never going** to transition successfully even when I move out. **Im never going** to be happy with the way I look or sound. **Im never going** to have enough friends to satisfy me. **Im never going** to have enough love to satisfy me. **Im never going** to find a man who loves me. (...)
11SA	i-you	Dearest darling **i want you** to know that you are the only one in my life **i love you** so much **i could not do without you** please forgive me i drove myself sick honey please beleave me **i love you** again and the baby honey don't be mean with me please I have lived fifty years since i met you **I love you** - **I love you**. Dearest darling i **love you i love you**. (...)
10SA	s/he-tell	(...) I am sorry to tell you this but **Lizzie told** you that I was drinking again. (...) left her in my bed and when she got up that was Mon **She went home told** me. (...) The **one that told** me didn't no Lizzie was my wife. (...) **He seen it and told** her she could do better then that. (...) I **told her** Sun. (...)
81SA	feel-i	(...) By day's end **I felt** that there is no way out. (...) All she can do it nitpick about how **I need to feel small** + helpless - yes **I do feel** that way but it doesn't help me to get back on my feet quickly. (...) **I feel angry** + that's better than feeling empty. (...) I rode bocycle for 1 mile Monday **I feel just awful**. (...)
4SA	i-leave	(...) so I am taking the only way out and **I leave** everything which has all been acquired since we were married to you my darling wife (...) **I leave** everything of value of any kind or nature inlcuding real estate (...)
96SA	i-know	(...) **I know** the names of a couple of them who are resident in the area (...)
9SA	give-s/he	(...) I **William Collins give ever thing to Henry Crawford** my car and what ever I have. (...) William Collins **Jane give all of my possessions to Elinor** and dont want Lizzie to attend my funeral. (...) The vW License[] should be retrieved from (auto shop) across the street and **given to Jane** when she turns 30 (...)
47SA	call-you	(...) I **called your** house but you unplugged your phone You would never answer my calls at your work. (...) **You could at least call** me (...) **you won't even call** me. (...)
75SA	i-want	(...) This is the way **I want** it (...) Most importantly Mom **I want** you to understand I must be cremated not buried an **I do not want** any kind of service (...) **I don't want** those assholes Jane & Christopher to get my car. (...) Mom **I want** you to contact Christopher for me. (...)

Table 3. Examples of sentences consisting of relations between given concepts. Original spelling was kept.
